# Mutation Spectrum in the *CACNA1A* Gene in 49 Patients with Episodic Ataxia

**DOI:** 10.1038/s41598-017-02554-x

**Published:** 2017-05-31

**Authors:** Cèlia Sintas, Oriel Carreño, Noèlia Fernàndez-Castillo, Roser Corominas, Marta Vila-Pueyo, Claudio Toma, Ester Cuenca-León, Isabel Barroeta, Carles Roig, Víctor Volpini, Alfons Macaya, Bru Cormand

**Affiliations:** 1 Departament de Genètica, Microbiologia i Estadística, Facultat de Biologia, Universitat de Barcelona, Barcelona, Catalonia Spain; 2 Centro de Investigación Biomédica en Red de Enfermedades Raras (CIBERER), Madrid, Spain; 3 Institut de Biomedicina de la Universitat de Barcelona (IBUB), Barcelona, Catalonia Spain; 4 Institut de Recerca Sant Joan de Déu (IR-SJD), Esplugues, Catalonia Spain; 5 Pediatric Neurology Research Group, Vall d’Hebron Research Institute, Universitat Autònoma de Barcelona, Barcelona, Catalonia Spain; 6 Department of Health and Experimental Sciences, Universitat Pompeu Fabra, Barcelona, Catalonia Spain; 7 Hospital del Mar Research Institute (IMIM), Barcelona, Catalonia Spain; 8 Neuroscience Research Australia, Sydney, New South Wales Australia; 9 School of Medical Sciences, University of New South Wales, Sydney, New South Wales Australia; 10 Psychiatric and Neurodevelopmental Genetics Unit (PNGU), Massachusetts General Hospital, Boston, Massachusetts United States of America; 11 Center for Psychiatric Research, Broad Institute of MIT and Harvard, Cambridge, Massachusetts United States of America; 12 Department of Neurology, Hospital de la Sta, Creu i St Pau, Barcelona, Catalonia Spain; 13 Department of Medicine, Universitat Autònoma de Barcelona, Barcelona, Catalonia Spain; 14 Centre de Diagnòstic Genètic-Molecular, Institut d’Investigació Biomèdica de Bellvitge (IDIBELL), l’Hospitalet de Llobregat, Barcelona, Catalonia Spain

## Abstract

Episodic ataxia is an autosomal dominant ion channel disorder characterized by episodes of imbalance and incoordination. The disease is genetically heterogeneous and is classified as episodic ataxia type 2 (EA2) when it is caused by a mutation in the *CACNA1A* gene, encoding the α_1A_ subunit of the P/Q-type voltage-gated calcium channel Ca_v_2.1. The vast majority of EA2 disease-causing variants are loss-of-function (LoF) point changes leading to decreased channel currents. *CACNA1A* exonic deletions have also been reported in EA2 using quantitative approaches. We performed a mutational screening of the *CACNA1A* gene, including the promoter and 3′UTR regions, in 49 unrelated patients diagnosed with episodic ataxia. When pathogenic variants were not found by sequencing, we performed a copy number variant (CNV) analysis to screen for duplications or deletions. Overall, sequencing screening allowed identification of six different point variants (three nonsense and three missense changes) and two coding *indels*, one of them found in two unrelated patients. Additionally, CNV analysis identified a deletion in a patient spanning exon 35 as a result of a recombination event between flanking intronic Alu sequences. This study allowed identification of potentially pathogenic alterations in our sample, five of them novel, which cover 20% of the patients (10/49). Our data suggest that most of these variants are disease-causing, although functional studies are required.

## Introduction

Episodic ataxia type 2 (EA2, MIM #108500) is a rare autosomal dominant ion channel disorder caused by mutations in the *CACNA1A* gene and characterized by episodes of midline cerebellar disturbance manifesting as ataxia, imbalance, vomiting, oscillopsia^1^ and interictal nystagmus; progressive ataxia can eventually develop^2^. EA2 has a wide phenotypic spectrum which includes paroxysmal neurological features other than ataxia. Around 50% of patients also experience migraine, and 80% suffer from rotational vertigo during the attacks^3^. Ataxia episodes last from hours to 2–3 days and are usually triggered by emotional stress, physical exercise, alcohol or caffeine. Onset usually occurs during the second decade of life, although later onsets have been reported^3, 4^. Acetazolamide administration can stop or diminish the frequency and severity of the attacks^5^.

*CACNA1A* encodes the pore-forming α_1_ subunit of the neuronal voltage-gated P/Q-type calcium channel (Ca_v_2.1), which is widely expressed in the central nervous system (CNS), especially in Purkinje cells and granule cells of the cerebellum. The Ca_v_2.1 channel is responsible for the coupling of calcium influx to vesicular exocytosis, mediating neurotransmission^6^. Since the first *CACNA1A* disease-causing variants were described in EA2^7^, over 80 EA2 alterations have been reported in the gene^3^. Several other neurological disorders are caused by pathogenic variants in *CACNA1A*, including familial hemiplegic migraine (FHM1, MIM #301011) and spinocerebellar ataxia type 6 (SCA6, MIM #183086), and the gene has also been related to other hemiplegic migraine (HM)-associated phenotypes like alternating hemiplegia of childhood^8^, acute striatal necrosis^9^, hemiplegia-hemiconvulsion-epilepsy^10^ or recurrent ischemic stroke^11^.

The vast majority of EA2-causing variants in the *CACNA1A* gene are predicted to cause loss of function of the channel, since nonsense, splicing and indel variants have been extensively reported. Furthermore, missense variants leading to decreased channel currents have been described^3, 4, 12, 13^ and over the past years deletions in *CACNA1A* have been reported in EA2 patients using quantitative approaches, such as Multiplex Ligation dependent Probe Amplification (MLPA)^14, 15^ or Quantitative Multiplex PCR of Short fluorescent Fragments (QMPSF)^16^. Functional studies have been carried out to investigate the pathogenic mechanism of EA2 mutations by expressing Ca_v_2.1 channels carrying either missense or truncating *CACNA1A* changes in mammalian cells^17, 18^ and *Xenopus* oocytes^19^. Two main hypotheses, negative dominance and haploinsufficiency, have been tested.

In this study we aimed to perform an extensive mutation analysis of the *CACNA1A* gene in 49 unrelated patients with episodic ataxia by means of sequencing and CNV analyses to identify both disease-causing point variants and structural variants.

## Results

### Patients

Ten out of 49 patients were found to harbour potentially pathogenic heterozygous *CACNA1A* variants (Table 1). Their clinical signs were prototypical for episodic ataxia. However, since most patients were adults when interviewed, complete information regarding their clinical presentation and the periods with higher attack frequencies was difficult to retrieve. Acetazolamide was generally effective in preventing the spells. Brain MRI documented vermian cerebellar atrophy in just one of the ten cases with molecular diagnosis.

**Table 1:** Clinical information and results from the *CACNA1A* mutational screening in episodic ataxia patients with identified variants.

Patient	Age of onset (years)	Frequency of the episodes (per month)	Duration	Trigger(s)	MRI	Other features	Variant				Restriction enzyme	PhyloP^c^/PhastCons^d^ scores	SIFT score^e^	PolyPhen-2 score^f^	Reference
							cDNA^a^	Exon	Protein^b^	Domain					
EP-004	infancy	NA	NA	NA	NA	ACZ responsive, MO, learning disability, motor clumsiness	c.749delG	5	p.G250Efs*60	DI, S5-S6 loop	+*Taq*I (mismatch)	5.84/1	—	—	—
432B	6	>1	15′-3 h	stress, fatigue	normal	ACZ responsive	c.G959A	6	p.W320*	DI, S5-S6 loop	+*Acc*I	5.78/1	—	—	—
A03_44	NA	NA	NA	NA	NA	FHM, interictal ataxia, nystagmus	c.C1502T	11	p.T501M^g^	DII, S1	+*Fat*I	4.32/1	Damaging (0.00)	Probably damaging (1.00)	24
A98_279	58	∼30	30′-1 h	NA	normal	episodic hand dystonia	c.G1913A	14	p.G638D	DII, S5-S6 loop	−*Aci*I	5.63/1	Damaging (0.03)	Probably damaging (1.00)	4
7A806	5	4	NA	NA	NA	MO	c.2042-43delAG	16	p.Q681Rfs*100	DII, S5-S6 loop	-*Dde*I	—	—	—	21–24
474	8	1–4	3–6 h	NA	deep WM hyperinte-nsities	worsen on CBZ and PHT, ACZ responsive	c.5253-2259_5403+ 1135del	35	p.S1753Cfs*2	DIV, S5-S6 loop	—	—	—	—	—
335a	<1	NA	48 h	NA	normal	migraine, visual aura	c.T5547A	37	p.Y1849*	Cytoplasmic tail	−*Psi*I	4.33/1	—	—	—
340	4	NA	NA	exercise, fatigue, emotional stress	temporal arachnoidal cyst	congenital squint, nystagmus, ACZ responsive	c.C5569T	37	p.R1857*	Cytoplasmic tail	+*Bsp*HI (mismatch)	3.33/1	—	—	20
389A	2	8–12	6–8 h upon sleep	stress, exercise, coffee, tea	mild cerebellar atrophy	improves on ACZ, MO, interictal ataxia, nystagmus	c.C5569T	37	p.R1857*	Cytoplasmic tail	+*Bsp*HI (mismatch)	3.33/1	—	—	20
349A	1.5	1–2	30′	stress	normal	nystagmus	c.C6665T	46	p.P2222L^g^	Cytoplasmic tail	−*Fau*I	2.83/1	Tolerated (1.00)	Benign (0.06)	—

MRI: Magnetic Resonance Imaging; NA: not available; ACZ: acetazolamide; MO: migraine without aura; FHM: Familial Hemiplegic Migraine; WM: white matter; CBZ: carbamazepine,; PHT: phenytoin; D: domain; S: segment. ^a^Reference sequence for cDNA nomenclature: NM_001127221 (nucleotide c.279A corresponding to the initiation codon, ATG). ^b^Rererence sequence for protein nomenclature: NP_001120693. ^c^PhyloP score: Positive scores indicate sites that are predicted to be conserved, whereas negative scores indicate sites predicted to be fast-evolving. ^d^PhastCons score: It ranges from 0 (non conserved amino acid positions) to 1 (highly conserved amino acid positions). ^e^SIFT score: It ranges from 0 to 1. The amino acid substitution is predicted as damaging if the score is <=0.05, and tolerated if the score is >0.05. ^f^PolyPhen-2 score: It ranges from 0 to 1. The aminoacid substitution is predicted as probably damaging if the score is >0.85, possibly damaging (score is between 0.15 and 0.85) or benign (score < 0.15). ^g^Variants p.T501M and p.P2222L have the rs codes rs121908240 and rs778551911, respectively.

### Sequencing analysis of *CACNA1A*

**Figure 1:**
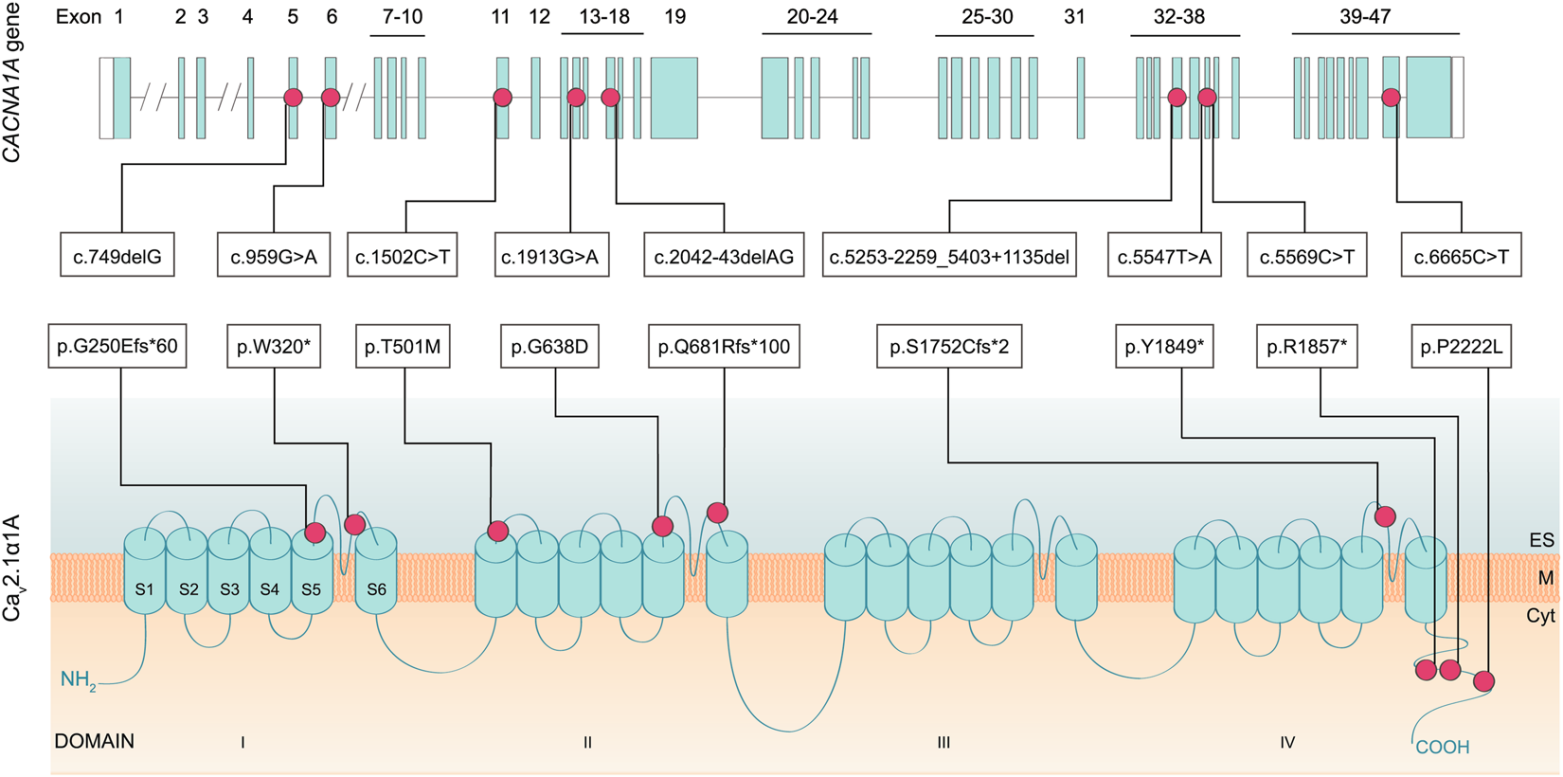
*CACNA1A* gene and protein structure with the identified pathogenic variants. *CACNA1A* gene structure (top figure), with boxes indicating exons. Protein structure of the Ca_v_2.1α_1A_ subunit (bottom figure). The genetic variants reported in this work are indicated by dots. Cyt: cytoplasm; M: cytoplasmic membrane; ES: extracellular space; S: Segment. Reference sequence for cDNA nomenclature: NM_001127221 (nucleotide c.279A corresponding to the initiation codon, ATG). Rererence sequence for protein nomenclature: NP_001120693.

**Figure 2:**
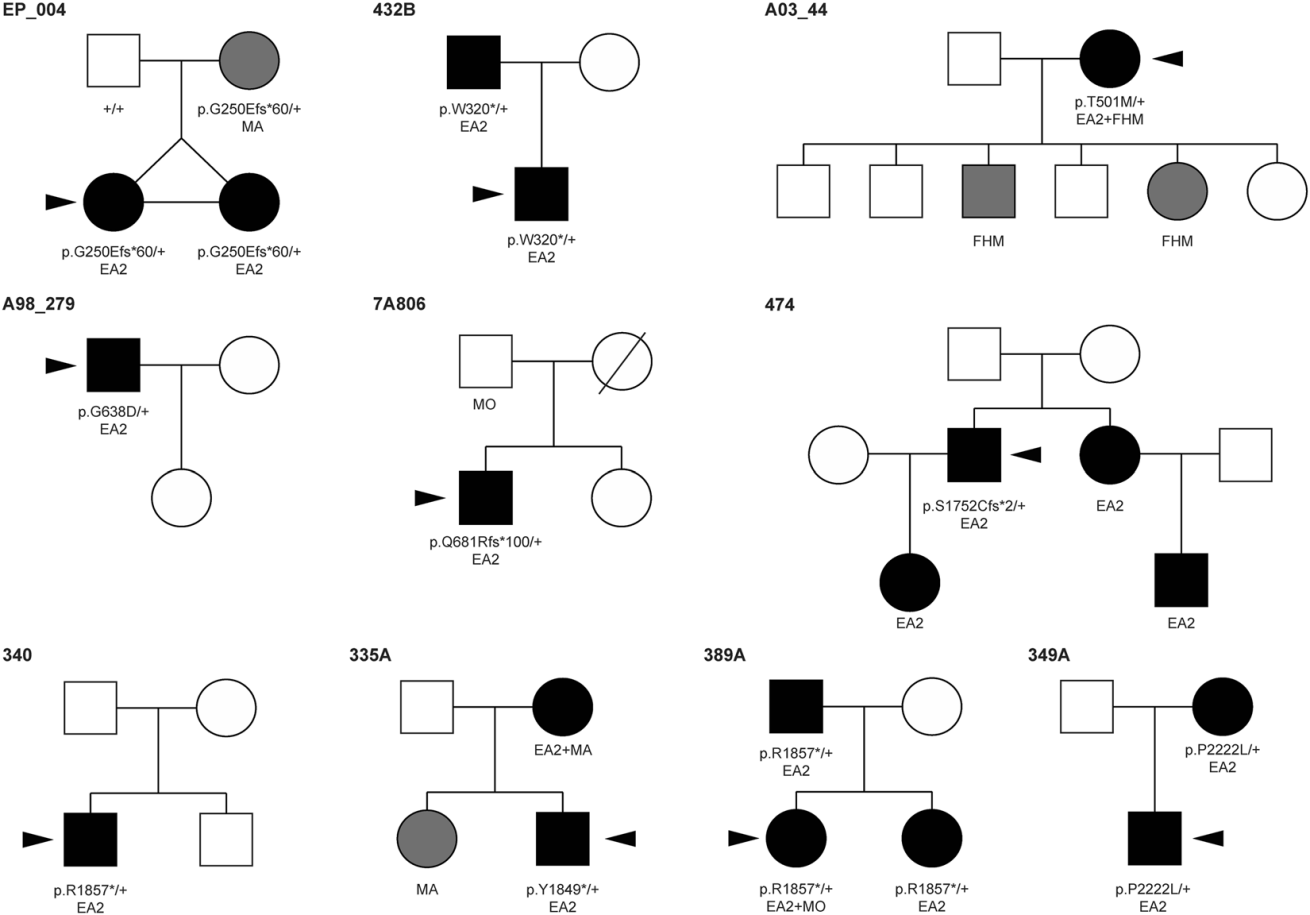
Pedigrees from individuals with identified changes. The code on top of every pedigree corresponds to the proband individual, indicated by a black arrow. Affected individuals are denoted by solid symbols; episodic ataxia is indicated in black and other phenotypes in gray; squares indicate males and circles indicate females. Clinical characteristics are indicated below each individual (EA2: episodic ataxia type 2; FHM: familial hemiplegic migraine; MO: migraine without aura; MA: migraine with aura). Gene variant carrier status is indicated below individuals. A complete screening of the *CACNA1A* gene was performed by Sanger sequencing and CNV analysis only in the probands (indicated by an arrow), whereas in the other family members where the genotype is indicated, only the variant identified in the proband was tested. In those individuals where the genotype is not shown, DNA was not available for analysis.

Sanger-sequence analysis of the *CACNA1A* gene in a cohort of 49 Spanish individuals diagnosed with episodic ataxia allowed the identification of eight heterozygous variants in nine patients (Figs 1 and 2). These included three missense changes, three nonsense variants leading to premature stop codons (one of them found in two unrelated patients) and two *indels* causing frame shift (Table 1). All variants were confirmed by restriction analysis (Table 1). Four of them are novel and four have been reported previously. None of the variants were present in a screening of 200 Spanish control individuals and all but two were absent from two different exome databases, one specific for the Spanish population (http://csvs.babelomics.org), including 576 subjects, and the ExAC resource (http://exac.broadinstitute.org) that includes more than 60,000 individuals. The p.T501M variant was found once in ExAC and p.P2222L was detected in four subjects in the same database, whereas the rest of the changes were not present in either dataset. In the nine families where a rare *CACNA1A* variant was identified, seven relatives were also screened for the particular change identified in the index case (Fig. 2). These relatives, five with episodic ataxia, one with migraine with aura and one healthy subject, belong to four of the families. The six relatives with a paroxystic phenotype bear the same mutation as the index case, whereas the healthy individual is not a carrier. In all four pedigrees the variant was transmitted, so no *de novo* changes were revealed by our analyses.

Two of the nonsense variants, p.W320* and p.Y1849*, are described here for the first time. The first one is located in the extracellular loop between S5 and S6 from domain I, maintaining only the N-terminus and part of the first domain of the protein (Fig. 1). Variant p.Y1849*, in contrast, leads to the truncation of the cytoplasmic tail, leaving out of the subunit the calcium binding domain (Fig. 1). Two other patients in our series carried the p.R1857* change, reported previously by other authors^20^.

We identified two coding *indels* leading to frame shifts and predicting truncated proteins. The novel genetic variant c.749delG (p.G250fs*60) is located in the S5-S6 extracellular loop from domain I, the same domain where the p.W320* change is located. The second one, c.2042-43delAG (p.Q681Rfs*100) produces a frame shift in domain II, also in the extracellular loop S5-S6, with a premature stop codon 100 codons downstream. This variant had been described in previous works^21–24^.

Finally, three missense variants were identified. Two variants had already been reported by our group, but they were added to the present study to provide a complete view of our episodic ataxia series: Patient A98_279 carries variation p.G638D^4^ and patient A03_44, diagnosed with both episodic ataxia and FHM carries the p.T501M change^25^. Both reports were accompanied by functional assays. The third variation, p.P2222L, described here for the first time, is located in a poly-proline region in the cytoplasmic tail of the protein.

### CNV analysis of *CACNA1A*

**Figure 3:**
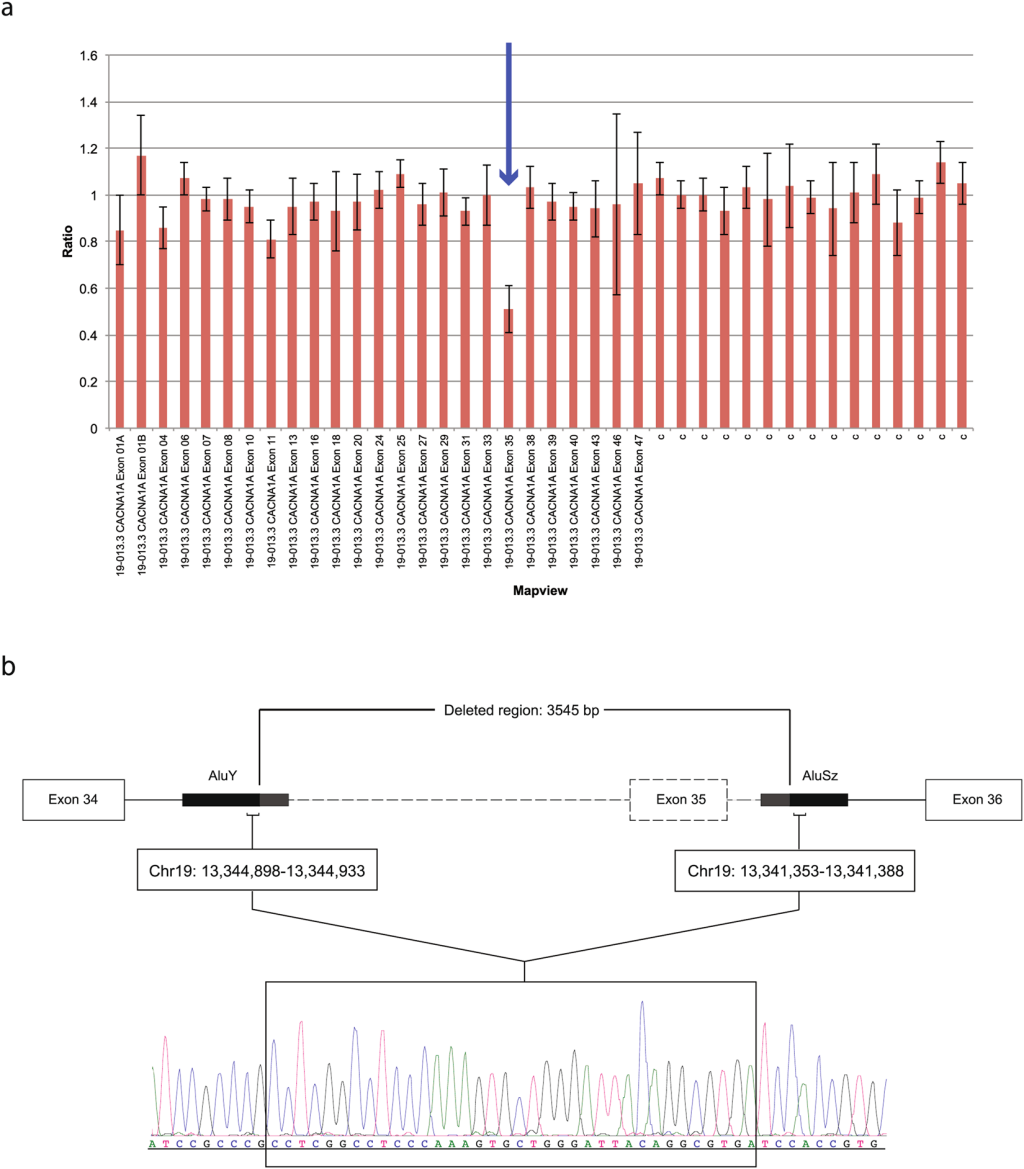
CNV studies in *the CACNA1A* gene of patient 474. (**a**) Results from the MLPA analysis performed with Coffalyser. The deleted exon 35 is indicated with an arrow. C: control probe. (**b**) Deletion breakpoint mapping. Sequence chromatogram corresponding to the homologous fragment from AluY and AluSz sequences. The identical shared sequence by the 5′ and 3′ Alu elements located in introns 34 and 35 that mediated the recombination is framed. Location of fragments taken from UCSC Genome Browser on Human Feb. 2009 Assembly (GRCh37/hg19).

The study of potential structural variants in the *CACNA1A* gene by MLPA allowed the identification of a deletion of the entire exon 35 (Fig. 3) in patient 474, which was confirmed by QMPSF.

In order to map the deletion breakpoint, a genomic region covering exons 34 to 36 was PCR-amplified and sequenced. A recombination occurred between two identical fragments located within Alu sequences in introns 34 (AluY) and 35 (AluSz), proximal and distal from exon 35 (Fig. 3), resulting in a 3.5-kb deletion. This novel deletion disrupts the coding frame from amino acid position 1,752 and is predicted to lead to a premature truncation only one residue downstream (p.S1752Cfs*2) in the extracellular S5-S6 loop of domain IV.

### Nonsense mediated mRNA decay (NMD) assay

An NMD assay was performed from skin fibroblasts of patient 432B, bearing a heterozygous p.W320* stop variant. Sanger sequencing and RFLP analysis were performed and the mutated allele was shown to be present both in cells treated with cycloheximide (an inhibitor of mRNA decay) and those untreated. Gel quantification showed that the transcript produced from the mutated allele accounts for 55% of the total amount of product), indicating that this process does not trigger degradation of the mRNA encoding the truncated protein (Supplementary Fig. S1).

## Discussion

Here we report an exhaustive screening of the *CACNA1A* gene in a large sample of patients with episodic ataxia, which was addressed through Sanger sequencing and CNV analyses. We identified nine different potentially pathogenic genetic variants in 10 of the 49 patients studied, five of which are novel (Table 1). This represents resolution of 20.4% of our patients sample, a percentage that is similar to others reported elsewhere^1, 26^. All these variants map to exons that are present in the main *CACNA1A* isoform (NM_001127221), which is expressed in brain, with highest expression in the cerebellar cortex, according to the public transcriptomic datasets Allen Brain Atlas (http://human.brain-map.org) and Human Brain Transcriptome (http://hbatlas.org/pages/hbtd). Although most of the previously reported changes in episodic ataxia type 2 (EA2) are point variations, deletions encompassing one or more *CACNA1A* exons have also been described^14–16, 27^. For this reason, in addition to sequencing exons, splice sites, branch points and the promoter region of the gene, we used two complementary quantitative approaches, MLPA and QMPSF, to extend the mutational screening of deletions and duplications within the gene. MLPA and QMPSF allowed inspection of 40 out of 48 *CACNA1A* exons (Supplementary Fig. S2). Thus, an exhaustive genetic diagnostic protocol consisting of two sequential approaches offered a wider and more comprehensive view of the genetic background of EA2.

We identified a total of three nonsense and three missense variants, two *indels* (one of them found in two patients) and a deletion encompassing exon 35 of the gene. From these, six were predicted to truncate the protein, either by introducing a stop codon (p.W320*, p.Y1849* and p.R1857*) or by causing a frame shift (p.G250Efs*60, p.Q681Rfs*100 and p.S1752Cfs*2). Disrupting variants that result in a loss of function of the Ca_v_2.1 calcium channel are the most commonly reported changes in EA2 patients. Functional analyses of the truncated CACNA1A subunit have showed diminished or null activity of the channel for EA2 mutations^18^. Thus, although truncating mutations have usually shown to cause a loss of function of the mutated subunit, the underlying pathophysiological mechanism that causes the EA2 phenotype remains still unclear. Two hypotheses have been proposed: haploinsufficiency, supported by the finding of nonsense mediated RNA decay (NMD)^15, 28^, and a dominant negative effect of the mutated subunit. The latter is more generally accepted as the major mechanism on the basis of functional studies that support an altered interaction between the WT and the mutated allele that would retain the complex in the endoplasmic reticulum, affecting protein trafficking and activating the proteasome response^17, 29, 30^. The dominant negative effect of the interaction may require the presence of the N-terminus of the protein in the mutant form, leading to a suppression of the Ca_v_2.1 channel expression due to the interaction between truncated and full-length subunits^18^. All six truncating changes reported here are located beyond the N-terminus of the channel and so are candidates to undergo dominant negative effects.

In our study, since skin fibroblasts from patient 432B (p.W320*) were available, the hypothesis of haploinsufficiency caused by a possible degradation of the mutated mRNA by NMD could be tested. No mRNA degradation was observed for this particular genetic variant in these cells (see Supplementary Fig. S1), although this result may not reflect what actually occurs in brain. NMD could not be tested in the rest of the patients from our collection, as biological samples were not accessible for analysis.

Three heterozygous missense variants (p.T501M, p.G638D and p.P2222L) were identified in three patients. The effect of missense variants are not easy to predict functionally. Two of the variants that we describe in this study, p.G638D and p.T501M, had been reported in previous works from our group, where functional analyses were performed^4, 25^. Variant p.G638D showed a loss of function of the channel, in agreement with the most commonly described effect for EA2-causing mutations. On another hand, the patient bearing variant p.T501M, a change reported previously in EA2^24^, presents with a phenotype that combines EA2 and HM. The functional analysis of this missense change revealed a gain of function of the channel, a pathogenic mechanism typical of HM rather than EA2. There are other reported cases where related phenotypes overlap, such as HM with progressive ataxia^31^ or EA2 with migraine^24^. The third missense variant, p.P2222L, was not functionally characterized. Although this variation was also detected in the mother of the index case, also with episodic ataxia (Fig. 2), neither SIFT^32^ nor PolyPhen-2^33^ predicted a damaging effect, whereas PhyloP^34^ scored it as a moderately conserved residue, and PhastCons^35^ as a highly conserved one (Table 1). The variant, rare, was not found in a set of around 600 exomes of the CIBERER Spanish Variant Server nor among 200 healthy Spanish individuals screened by us, and it was found at heterozygosity in only 4 out of 6,000 subjects from the ExAC database (although this last figure should be taken with caution as the relevant position is covered only in 10% of the individuals, possibly indicating low-quality sequences). These indicators (a rare conserved variant that cosegregates with the disorder but has no favourable damaging effect predictors) are contradictory and thus a functional test is needed to shed light on the impact of the p.P2222L change.

Taking together this and other studies, no specific prevalent variants are found in EA2, which results in large allelic heterogeneity. However, there are some regions in the *CACNA1A* gene that are found to be more frequently mutated in EA2 patients and also in other patients presenting ataxic features (cerebellar ataxia)^26^. In our study, five out of nine variants are located in the S5-S6 extracellular loop of different domains (I, II and IV) (Fig. 1). Many disease-causing changes, both nonsense and missense, in EA2 seem to be preferentially located in these areas. Therefore, this S5-S6 linker may represent a key region that influences the proper functionality of the subunit. This effect has also been seen in mouse models bearing changes in this region, which present a mild ataxia phenotype^36^. Only one variant in our study, p.T501M, present in a patient with EA2 and HM, is located in a transmembrane domain (DII-S1) that belongs to the voltage sensor part, affecting both activation and inactivation of the channel^25^.

Three other variants identified here are located in the C-terminal tail. The two nonsense variants p.Y1849* and p.R1857* are in exon 37, within the EF-hand responsible for calcium binding, so the IQ-like CaM interaction domain (IQ) and the Calmodulin Binding Domain (CBD)^36^, located donwstream, are also lost. Finally, although the missense variant p.P2222L is located downstream from these interaction regions (EF-hand and calmodulin binding domains), it may affect the conformation of the tail and disturb the interaction between the CACNA1A subunit and other elements that bind the C-terminal region, such as auxiliary β-subunits^37, 38^, leading to an impaired function of the channel.

Genotype-phenotype correlations were not apparent in our cohort: all pathogenic variants described in this study produced comparable EA2 phenotypes regardless of their molecular nature or they location in transmembrane or cytoplasmic tail protein domains. Of note, two patients (cases 340 and 389A) displayed the same variant p.R1857*; in that particular instance the clinical presentation was similar in the early age of onset and presence of interictal cerebellar signs.

In summary, we have identified nine potentially disease-causing variants in ten patients with episodic ataxia. However, there is still a significant proportion of subjects with this phenotype that bear no mutations in the *CACNA1A* gene. Although there might be some degree of missing allelic heterogeneity in *CACNA1A*, possibly other genes are involved in the disorder in our sample, which may be uncovered by means of next generation sequencing approaches. Indeed, genes other than *CACNA1A* have previously been involved in episodic ataxia, including *KCNA1* (episodic ataxia type 1, EA1)^39^, *CACNB4* (EA4)^40^ and *SLC1A3* (EA6)^41^. More recently, other potential EA genes have been reported, including *SCN2A* ^42^, *FGF14* ^43^ and *SLC2A1* ^44^. With the exception of *CACNA1A* and *KCNA1*, the rest have been found mutated in only one or a few patients, indicating that the number of genes involved in this neurologic phenotype may be high.

## Methods

### Patients

All 49 patients were diagnosed with episodic ataxia on clinical grounds by expert neurologists. Central to the diagnosis was eliciting a history of recurrent paroxysmal attacks of ataxia, vertigo, and nausea or vomiting typically lasting minutes to days in duration. Additional supporting criteria were (i) the presence of interictal ataxia and nystagmus; (ii) a history of the attacks being triggered by exercise, emotional stress, alcohol, caffeine, fever, or heat; (iii) reduction of attack frequency/severity by acetazolamide; (iv) absence of myokymia and (v) a family history consistent with autosomal dominant inheritance.

### Sampling and mutation screening

Peripheral blood samples were collected from all probands and genomic DNA was isolated using a standard salting-out method^45^. All 48 exons, splice sites and branch points from the *CACNA1A* gene were sequenced. The promoter (894 bp upstream from the translation initiation codon) and the 3′UTR region, containing exon 48, were also screened as previously described^25, 46^. All variants were assessed by bidirectional sequencing and confirmed by restriction fragment length polymorphism (RFLP) analysis. Two hundred control individuals were screened by Sanger sequencing, and the presence of the identified mutations was investigated at the Exome Aggregation Consortium dataset (ExAC, http://exac.broadinstitute.org) and the CIBERER Spanish Variants Server (http://csvs.babelomics.org). Seven relatives from four of the families with identified *CACNA1A* mutations were also screened by Sanger sequencing and RFLP analysis. Variant nomenclature follows Human Genome Variation Society (HGVS) guidelines (http://www.hgvs.org/mutnomen/recs-DNA.html) and refers to the *CACNA1A* cDNA sequence NM_001127221 (protein sequence NP_001120693), with nucleotide c.279A corresponding to the initiation codon (ATG).

### CNV analysis

Multiplex Ligation-dependent Probe Amplification (MLPA) was the first approach used for the CNV analysis. We used the MLPA *CACNA1A* kit SALSA-P279-A2 (MRC Holland, Amsterdam) that contains 25 probes covering 24 exons of the *CACNA1A* gene. This test was performed according to the manufacturer’s instructions. Quantitative Multiplex PCR of Short fluorescent Fragments (QMPSF) was used as a complementary approach to cover most of the exons that were not covered by the MLPA assay. Four sets of probes targeting 16 additional exons were designed. For five additional exons, since they were located <1 kb from the ones targeted by QMPSF or MLPA and no repeat sequences were present between them, deletion risk was considered as very low. A reference fragment from the *RNF20* gene was co-amplified in each multiplex. A new set of probes was designed to confirm the deletion identified by MLPA in one of the patients, containing the deleted exon and the flanking ones.

The MLPA analysis software Coffalyser v8 was used to evaluate the possible presence of CNVs, considering deletion when the ratio was under 0.7 and duplication when it was over 1.3. QMPSF data were analyzed using the PeakScanner™ v1.0 software (Applied Biosystems), and the final ratios for each exon were expressed using the following formula: (height of the peak corresponding to the tested fragment for the patient/height of the peak corresponding to *RNF20* for the patient)/(height of the peak corresponding to the tested fragment for the average of controls/height of the peak corresponding to *RNF20* for the average of controls). We considered a deletion when the ratio was under 0.6 and duplication when it was over 1.4. More details about the design and analysis are provided in Supplementary Fig. S2. PCR conditions and sample analysis procedures are available upon request.

In order to map the breakpoint of the deletion spanning exon 35, we PCR-amplified and Sanger-sequenced a genomic region containing exons 34 to 36 from the carrier patient (primers and conditions available upon request).

### Nonsense-mediated mRNA decay (NMD) assay

Only in one case biological material was available to perform NMD assays. Fibroblast primary cultures from a control individual and from patient 432B (bearing the nonsense variant p.W320*) were obtained from skin biopsies, cultured in a monolayer at 37 °C under 5% CO_2_ in T25 flasks (Greiner Bio-One, North America, Inc.) with DMEM medium (Sigma-Aldrich, Steinheim, Germany) containing 12% fetal bovine serum (FBS) (Gibco, Invitrogen Life Technologies, Heidelberg, Germany). After three weeks of maintenance, fibroblasts were tripsinized and cultured in 35mm dishes with DMEM medium containing 10% FBS and 1% Penicillin-Streptomicine (Invitrogen Life Technologies, Heidelberg, Germany).

To proceed with the NMD assay, WT and mutated cells were then cultured in a six well-plate each, and three replicates were treated with cycloheximide, a NMD inhibitor, at 1mg/ml during 6 hours.

RNA extraction from fibroblasts and reverse transcription polymerase chain reaction (RT-PCR) were performed using QIAshredder (Qiagen, Hilden, Germany) and High Capacity cDNA kit (Applied Biosystems) and following the manufacturer’s instructions. A segment of the *CACNA1A* cDNA including exon 6 was amplified in all samples. PCR products were sequenced and digested with *Acc*I, enzyme that cuts the fragment bearing the pathogenic variant. The PCR products were electrophoresed on a 2% agarose gel, stained with EtBr and quantified using the *ImageJ* software (https://imagej.nih.gov/ij).

All methods were performed in accordance with the relevant guidelines and regulations. This study was approved by the local Ethics Committee, the Institutional Review Board of the University of Barcelona (IRB00003099), and informed consent was obtained from all adult subjects, children, and their parents according to the Helsinki declaration.

## Electronic supplementary material

Supplementary information
