# Supplementary material for: Mutation Spectrum in the CACNA1A Gene in 49 Patients with Episodic Ataxia
Source: Sci Rep. 2017 May 31;7:2514. doi: 10.1038/s41598-017-02554-x (PMC5451382; doi:10.1038/s41598-017-02554-x)

**MUTATION SPECTRUM IN THE *CACNA1A* GENE IN 49 PATIENTS WITH EPISODIC ATAXIA**

Cèlia Sintas, Oriel Carreño, Noèlia Fernàndez-Castillo, Roser Corominas, Marta Vila-Pueyo, Claudio Toma, Ester Cuenca-León, Isabel Barroeta, Carles Roig, Víctor Volpini, Alfons Macaya, Bru Cormand

**Supplementary Figure S1. Nonsense mediated mRNA decay (NMD) assay of mutation p.W320* (c.959G>A).** a) cDNA sequence chromatograms of *CACNA1A* exon 6 from fibroblasts of patient 432, bearing an heterozygous p.W320* mutation, treated with cycloheximide or not treated (indicated as 432-Chx and 432-Nt, respectively), and from WT fibroblasts (WT). The normal sequence of the amplified fragment is indicated on top. The changed position is highlighted in red and shaded. b) Discrimination of the two alleles by agarose gel electrophoresis of *CACNA1A* cDNA exon 6 PCR products digested with *Acc*I, which cuts the product bearing the mutated variant. Both the mutant and the WT alleles are equally represented in cycloheximide-treated and untreated p.[W320*];[=] fibroblasts, indicating that there is no decay of the mutant RNA. On the right side, the sizes of the lower bands of the molecular weight marker are indicated.


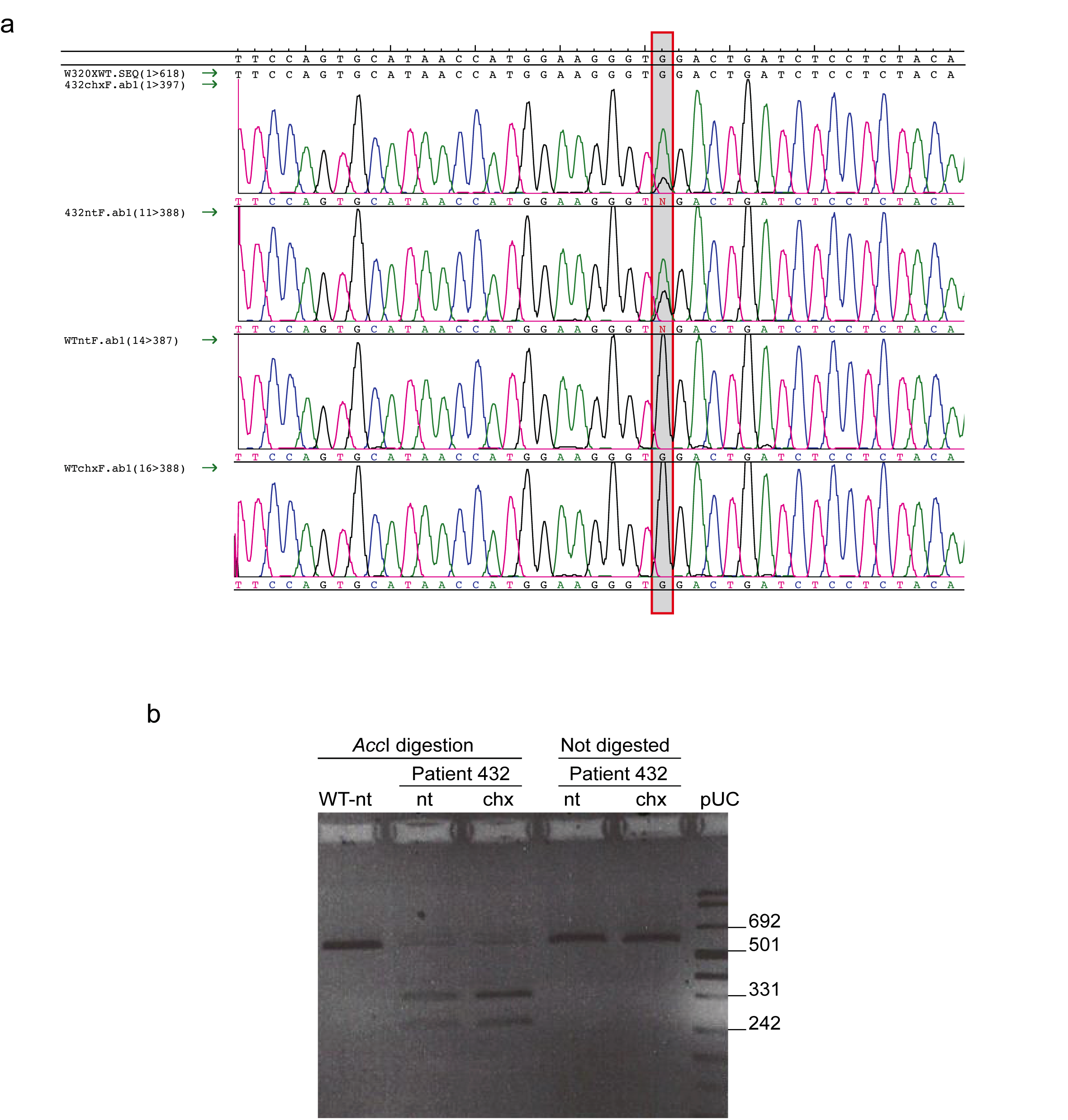
**a**

432-Chx

432-Nt

WT


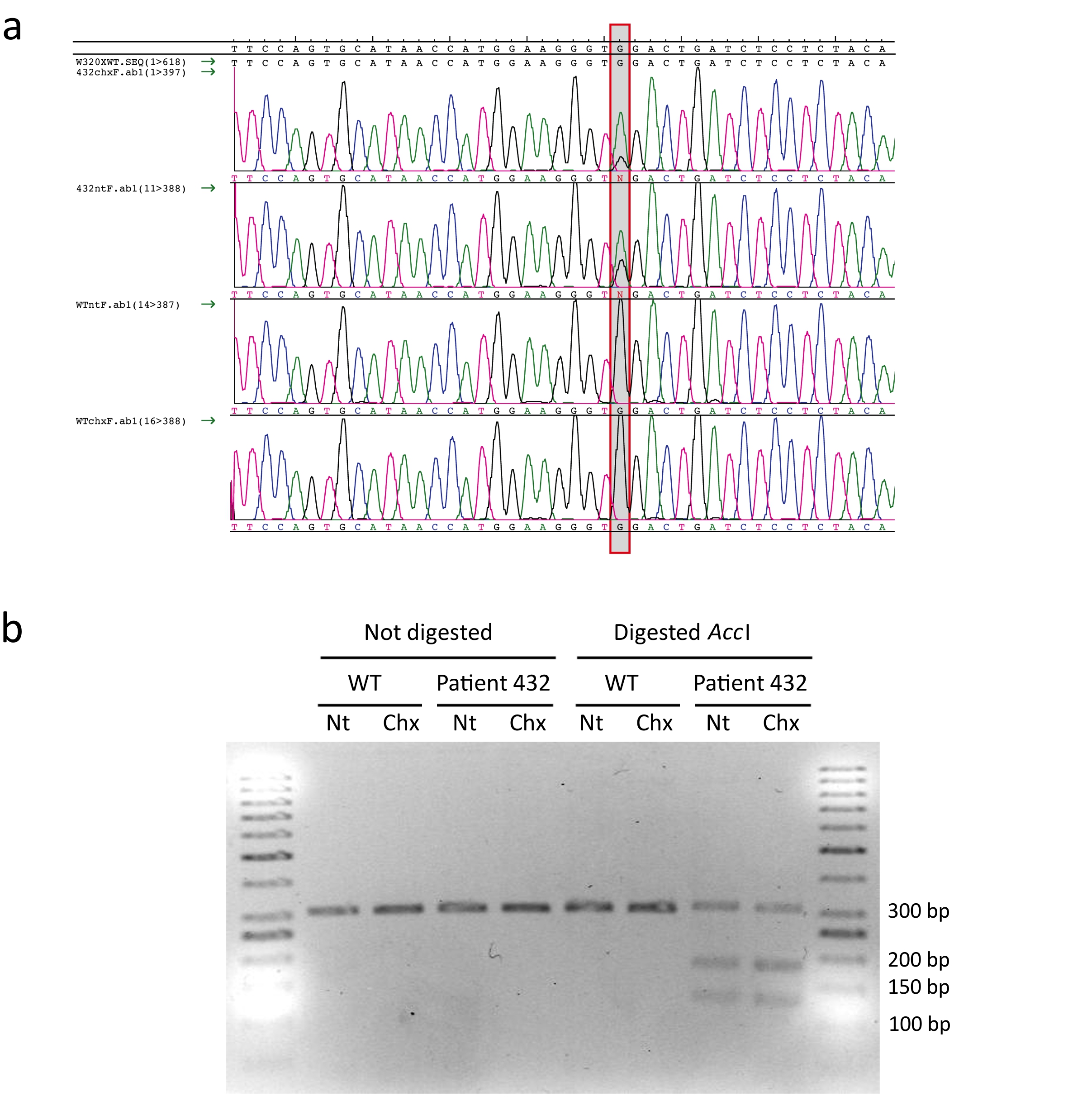
**b**

**Supplementary Figure S2. CNV study design for *CACNA1A* using Multiplex Ligation-dependent Probe Amplification (MLPA) and Quantitative Multiplex PCR of Short fluorescent Fragments (QMPSF) approaches**. Black symbols () indicate the exons inspected by each assay and grey symbols () indicate close exons that were not included. The MLPA kit allowed inspection of 24 exons. For QMPSF, we used four sets of primer pairs covering 16 additional exons. Two individuals with deletions spanning *CACNA1A* exons 20–47 and 32–47 were also included in every assay as positive controls.


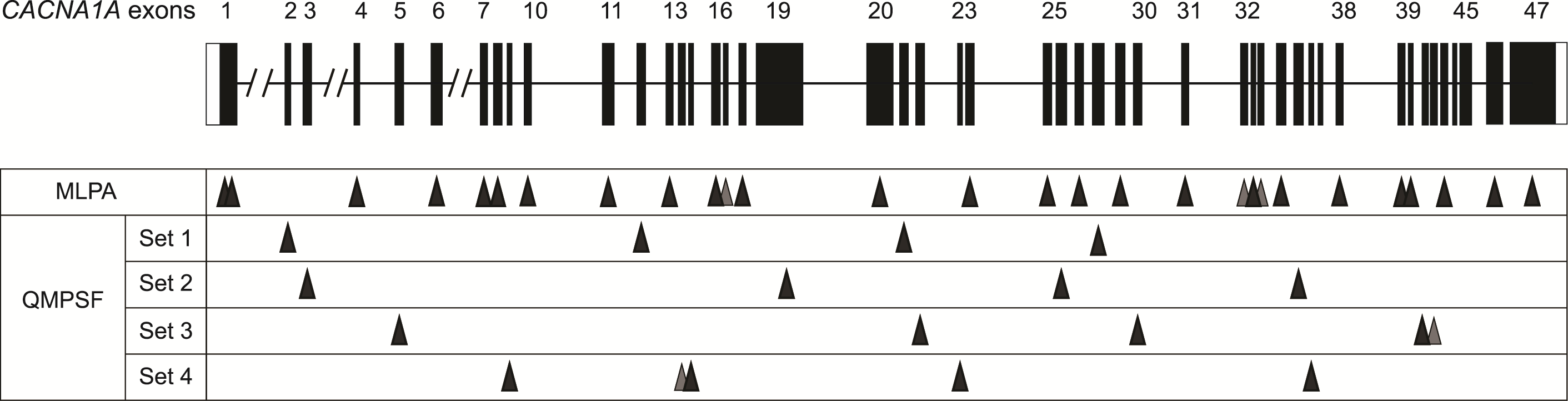

Supplement: Supplementary file 1 — Supplementary information [file 41598_2017_2554_MOESM1_ESM.doc]
